# Predicting functionally important SNP classes based on negative selection

**DOI:** 10.1186/1471-2105-12-26

**Published:** 2011-01-19

**Authors:** Mark A Levenstien, Robert J Klein

**Affiliations:** 1 1Program in Cancer Biology and Genetics, Memorial Sloan-Kettering Cancer Center, New York, NY 10065 USA

## Abstract

**Background:**

With the advent of cost-effective genotyping technologies, genome-wide association studies allow researchers to examine hundreds of thousands of single nucleotide polymorphisms (SNPs) for association with human disease. Recently, many researchers applying this strategy have detected strong associations to disease with SNP markers that are either not in linkage disequilibrium with any nonsynonymous SNP or large distances from any annotated gene. In such cases, no well-established standard practice for effective SNP selection for follow-up studies exists. We aim to identify and prioritize groups of SNPs that are more likely to affect phenotypes in order to facilitate efficient SNP selection for follow-up studies.

**Results:**

Based on the annotations available in the Ensembl database, we categorized SNPs in the human genome into classes related to regulatory attributes, such as epigenetic modifications and transcription factor binding sites, in addition to classes related to gene structure and cross-species conservation. Using the distribution of derived allele frequencies (DAF) within each class, we assessed the strength of natural selection for each class relative to the genome as a whole. We applied this DAF analysis to Perlegen resequenced SNPs genome-wide. Regulatory elements annotated by Ensembl such as specific histone methylation sites as well as classes defined by cross-species conservation showed negative selection in comparison to the genome as a whole.

**Conclusions:**

These results highlight which annotated classes are under purifying selection, have putative functional importance, and contain SNPs that are strong candidates for follow-up studies after genome-wide association. Such SNP annotation may also be useful in interpreting results of whole-genome sequencing studies.

## Background

With recent technological advances, genome-wide association studies are now a reality and offer terrific promise for localizing genes responsible for complex diseases. In fact, large-scale genome-wide association studies have successfully identified genetic loci related to a wide array of disorders. Currently, over 550 publications report putative associations that have been found by this approach [1]. A benefit of this relatively unbiased strategy is the potential to identify genes in pathways previously unconnected with the disease of interest. Examples include identification of complement factor H (*CFH*) in age related macular degeneration, *FGFR2 *in breast cancer, and *CDKN2A *as well as *CDKN2B *in type 2 diabetes [2-9]. Other successful studies have discovered loci related to a variety of disorders including cancer, autoimmune disease, and cardiovascular disease [10-12].

Genome-wide association studies often utilize multiple stages in which promising signals are identified in an initial stage and followed up in subsequent stages. The process of follow up includes fine-mapping a signal using the same cohort and replicating the finding in additional cohorts. In both cases, SNPs need to be prioritized for selection in follow up studies since it is currently impractical to experimentally test every SNP at our disposal for association with disease. Some groups advocate prioritizing SNPs in genes and/or coding regions over other SNPs during selection [13]. By not only emphasizing nonsynonymous SNPs but also applying computational methods to detect nonsynonymous variants with the greatest potential to disrupt protein function, others take this strategy a step further [14-19]. Such prioritization begins to address the problem of an unwieldy number of SNPs to evaluate. Unfortunately, these gene-centric approaches fail to focus on functionally important SNPs outside of annotated genes. As will be justified below, a more comprehensive prioritization scheme, which considers SNPs outside of protein-coding regions, is warranted.

A compelling reason to consider a broader approach for SNP selection is that often genome-wide association studies have produced highly significant signals that fall in regions far from any annotated protein-coding gene. It is likely that these findings are true positives since in many cases independent research groups have replicated these results. According to the National Human Genome Research Institute's catalog of published GWA studies, to date greater than 460 associations have been found in intergenic regions or in regions where the gene is unknown. Presumably, many more associations are located in genomic regions devoid of annotated genes but are not reported in the catalog [1]. One puzzling example is the region at 8q24, which has been implicated in studies of prostate, breast, and colorectal cancer [3,20-25]. The closest gene, *MYC*, is a viable candidate; however, it is more than 300 kb from the association signals. Another region implicated in multiple disorders is 9q21. Significant association signals in this region have been detected for myocardial infarction and type 2 diabetes and are 150 kb from the nearest genes [7-9,26-28].

Several researchers have applied approaches utilizing bioinformatics to investigate regions outside of coding regions for the existence of selective pressure. Chen *et al. *demonstrated significant negative selection in regions predicted as miRNA targets [29]. Similarly, others have shown negative selective pressure acting in predicted exon splicing enhancers, cis-elements, and introns [30-33]. Furthermore, negative selection has been identified in both conserved noncoding sequences and ultraconserved elements in the human genome although variations in these regions have not yet been linked to changes in phenotype [34-37]. A comprehensive study identifying genomic regions under negative selection utilizing a full array of the annotation currently available has not so far been undertaken. Such a study could provide the basis for a SNP prioritization scheme that extends beyond SNPs in coding regions.

Here we create a comprehensive scheme for prioritizing SNPs based on the likelihood that they are functional. Our approach involves identifying regions of the genome under negative selection. Since these regions appear intolerant of putatively deleterious alleles, they most likely harbor important functional features. We categorize the SNPs in the human genome based on the annotations available related to gene structure, cross-species conservation, and regulatory elements. By examining the distribution of derived allele frequencies within each class, we assess the strength of evidence for negative selection in each class. We advocate employing this information to weight the SNPs based on their class affiliations. This approach targets SNPs in genomic regions that display evidence of function, regardless of whether protein-coding genes are found in the region.

## Results

**Figure 1:**
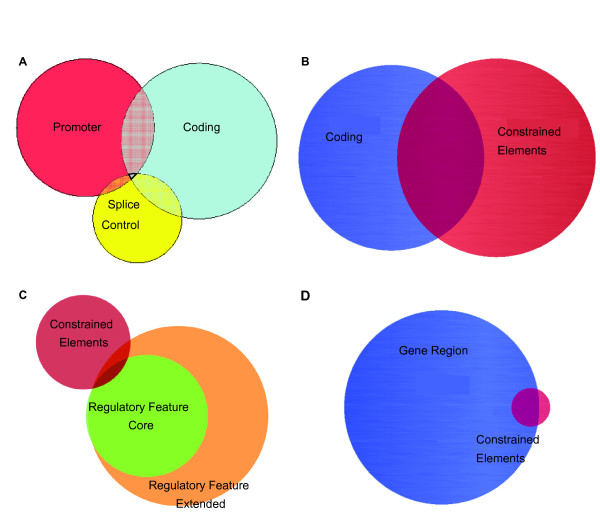
**Venn Diagrams displaying the relative number of genome-wide SNPs in several classes**. SNP markers are from Ensembl. A) Comparison of SNPs in the gene-centered annotations of promoter, coding, or splice control regions. B) Comparison between coding SNPs and evolutionarily constrained SNPs. C) Comparison between constrained elements and regulatory features. D) Comparison between constrained elements and genic regions.

We classified SNPs in the Ensembl database into 44 classes based on the annotation available as detailed in the Methods section (Table 1, Additional File 1). An examination of these classes revealed that they are not mutually exclusive (Figure 1). Interestingly, the vast majority of SNPs in constrained elements, regions of the genome that show a high level of cross species conservation, did not appear in either coding regions (Figure 1B) or the regulatory features constructed by experimentally derived regulatory attributes (Figure 1C). These observations emphasize that constrained elements represent a distinct class worthy of separate consideration from coding and regulatory elements. Likewise, the majority of SNPs in coding regions and regulatory features did not reside in constrained elements (Figure 1B and 1C).

**Table 1:** Representative classes considered in this study.

class name	number of SNPs	size of region (kb)	SNP frequency (SNPs/kb)
coding	132,562	34,215	3.87

promoter	104,439	24,844	4.20

splice site	41,932	11,621	3.61

constrained elements	152,158	71,977	2.11

regulatory features core	260,919	64,296	4.06

regulatory features extended	558,932	136,134	4.11

cisRED	9,491	2,815	3.37

miRanda	3,975	884	4.50

non-coding RNA genes	1,832	490	3.74

ancestral repeats	44,243	13,011	3.40

genome	11,307,522	3,022,647	3.74

SNP counts shown are for all SNPs in Ensembl.

To identify regions of the genome under purifying selection, we compared the distribution of derived allele frequencies (DAF) in each class with that of the genome as a whole and, in a confirmatory analysis, with that of ancestral repeats, a well-accepted model of neutral selection [38-42]. For the comparisons, we utilized the allele frequencies from three populations comprehensively genotyped by Perlegen--African Americans (AFR), European Americans (EUR), and Han Chinese from the Los Angeles area (CHN) [43]. An excess of low derived frequency SNPs in comparison to the genome (or ancestral repeats) implies a region is under negative selection.

We found evidence that suggests the existence of negative pressure for several classes. Seventeen classes were statistically significant (false discovery rate < 0.05) in the AFR population (Table 2 and Additional File 1). Similar results were observed for the EUR and CHN populations (Additional File 1). We emphasize the AFR population because it possesses greater genetic diversity and many fewer monomorphic SNPs, thereby increasing our power to detect negative selection. As expected, protein-coding gene related classes such as nonsynonymous, coding, and splice site classes resided on the list of significant classes. Constrained element classes and regions where four specific histone methylations have been detected (H3K79me3, H3K36me3, H3K4me2, and H3K4me3) appeared statistically significant. Several other regulatory elements, including microRNA binding targets (miRanda elements), are also worth noting.

**Table 2:** Classes under negative selection compared to the genome.

rank	class name	*p*-value	q-value
1	coding	<1 × 10^-8^	1.5 × 10^-3^

2	nonsynonymous	<1 × 10^-8^	3.1 × 10^-3^

3	constrained elements	<1 × 10^-8^	4.7 × 10^-3^

4	constrained elements minus coding	<1 × 10^-8^	6.3 × 10^-3^

5	constrained elements minus genes	<1 × 10^-8^	7.8 × 10^-3^

6	constrained elements 1 kb from genes	<1 × 10^-8^	9.4 × 10^-3^

7	regulatory features extended	<1 × 10^-8^	1.1 × 10^-2^

8	H3K36me3	<1 × 10^-8^	1.3 × 10^-2^

9	H3K79me3	<1 × 10^-8^	1.4 × 10^-2^

10	constrained elements 100 kb from genes	1.0 × 10^-8^	1.6 × 10^-2^

11	splice site	8.0 × 10^-4^	1.7 × 10^-2^

12	DnaseI	4.5 × 10^-3^	1.9 × 10^-2^

13	H3K4me3	5.1 × 10^-3^	2.0 × 10^-2^

14	H3K4me2	8.6 × 10^-3^	2.2 × 10^-2^

15	PolII	1.1 × 10^-2^	2.3 × 10^-2^

16	miRanda	1.5 × 10^-2^	2.5 × 10^-2^

17	cisRED	2.4 × 10^-2^	2.7 × 10^-2^

Classes with a statistically significant excess of low derived alleles when compared to the genome as a whole are shown. In order to adjust for the multiplicity of testing, we apply an FDR correction with α = 0.05. Only resequenced Perlegen SNP markers are included in this analysis to minimize ascertainment bias. For our comparisons, we rely on allele frequencies present in the AFR Perlegen population.

The above analysis rests on the assumption that the vast majority of the genome as a whole is under neutral selection and is therefore an appropriate benchmark for comparison. In order to validate this hypothesis, we utilized SNPs in ancestral repeats, a well-accepted model of neutral selection, in a second round of analyses for comparison [38-42]. The results from the comparisons with the ancestral repeats supported those from the comparisons with the genome as a whole. Six classes were statistically significant when compared to ancestral repeats for the Perlegen AFR population (Table 3 and Additional File 1). Again, the analyses using the EUR and CHN population allele frequencies showed similar results (Additional File 1). Similar to the comparison with the genome as a whole, the significant classes found in the comparison with ancestral repeats were the nonsynonymous and coding SNPs as well as all but one constrained elements classes. When comparing these results, it is important to recognize that the comparisons to the genome involved many more observations and, therefore, had a sizable power advantage to detect modest effects. Furthermore, we applied a less severe multiple test correction for the comparisons to the genome as a whole because we performed twelve fewer tests due to computational issues (see Methods).

**Table 3:** Classes under negative selection compared to ancestral repeats.

rank	class name	*p*-value	q-value
1	***nonsynonymous***	5.0 × 10^-8^	1.1 × 10^-3^

2	***constrained elements***	3.6 × 10^-5^	2.3 × 10^-3^

3	***constrained elements minus coding***	1.4 × 10^-4^	3.4 × 10^-3^

4	***coding***	1.2 × 10^-3^	4.5 × 10^-3^

5	***constrained elements minus genes***	3.0 × 10^-3^	5.7 × 10^-3^

6	***constrained elements 1 kb from genes***	3.2 × 10^-3^	6.8 × 10^-3^

7	H3K79me3	8.0 × 10^-3^	7.9 × 10^-3^

8	constrained elements 100 kb from genes	1.1 × 10^-2^	9.0 × 10^-3^

9	miRanda	3.4 × 10^-2^	1.0 × 10^-2^

10	H3K36me3	4.0 × 10^-2^	1.1 × 10^-2^

11	PolII	6.3 × 10^-2^	1.3 × 10^-2^

12	H3K4me2	7.1 × 10^-2^	1.4 × 10^-2^

13	cisRED	1.0 × 10^-1^	1.5 × 10^-2^

Classes with an excess of low derived alleles when compared to the ancestral repeats are shown. Bolded, italicized classes are statistically significant when we apply an FDR correction with α = 0.05. Only resequenced Perlegen SNP markers are included in this analysis to minimize ascertainment bias. For our comparisons, we rely on allele frequencies present in the AFR Perlegen population.

Some borderline significant classes (unadjusted *p *< 0.1) are also worth noting (Table 3). Interestingly, the H3K79me3 histone methylation class was extremely close to being significant. Albeit not as close as H3K79me3, the remaining constrained elements class, miRanda elements, and the H3K36me3 histone methylation class also seemed to provide strong evidence to support the findings from the initial comparison to the genome as a whole. Notably, several classes, including splice sites, Dnase I hypersensitivity sites, regulatory features extended (an Ensembl class summarizing the regulatory attributes), and H3K4me3, which appeared significant in the initial analysis did not appear significant or even borderline significant in the subsequent analysis involving the ancestral repeats. In particular, the regulatory features extended class may not appear significant in this subsequent analysis because this class is built from 24 distinct regulatory attributes - some displaying strong evidence of negative selection and other displaying little or no evidence of negative selection. While the regulatory features class as well as these other classes may still represent regions of the genome under negative selection and be functionally important, the confirmatory analysis utilizing ancestral repeats did not substantiate the initial findings.

As mentioned above, we observed evidence for negative selection among the SNPs in regulatory attributes, H3K79me3 and H3K36me3, as well as in constrained elements. We investigated the possibility that our observations were the result of an underlying hitchhiking effect or background selection. For the two regulatory attributes, we considered SNPs in random genomic regions comparably located to annotated genes (see Methods). In this approach, we compared the *p*-values reported above, "real *p*-values", with the *p*-values generated using the random genomic regions, "generated *p*-values". For both the H3K79me3 and the H3K36me3 experiments, we found that the "real" *p*-values were considerably smaller than all 100 "generated" *p*-values in comparisons to both the genome and ancestral repeats (Table 4). In addition, we applied a FDR test correction to the "real" *p*-values to adjust for the multiplicity of testing involved in performing 44 tests. Again, the FDR adjusted *p*-values were smaller than all 100 "generated" *p*-values in comparisons to both the genome and ancestral repeats (Table 4). These results suggest that a hitchhiking effect or background selection cannot account for the observed negative selection in the regions containing these regulatory attributes.

**Table 4:** *P*-values and FDR adjusted *p*-values for the analyses involving regulatory attributes H3K79me3 and H3K36me3.

	H3K79me3		H3K36me3	
	**ancestral repeats**	**genome**	**ancestral repeats**	**genome**

*p*-value (rank)	8.00 × 10^-3 ^(1)	1.00 × 10^-8 ^(1)	4.00 × 10^-2 ^(1)	1.00 × 10^-8 ^(1)

FDR adjusted *p*-value (rank)	5.03 × 10^-2 ^(1)	4.40 × 10^-7 ^(1)	1.76 × 10^-1 ^(1)	4.40 × 10^-7 ^(1)

Ranks for each "real" *p*-value are relative to a set of "generated" *p*-values produced by performing tests of evidence of selection on sets of SNPs in random genomic regions comparable, in size and proximity to annotated genes, to the regulatory feature under consideration.

**Figure 2:**
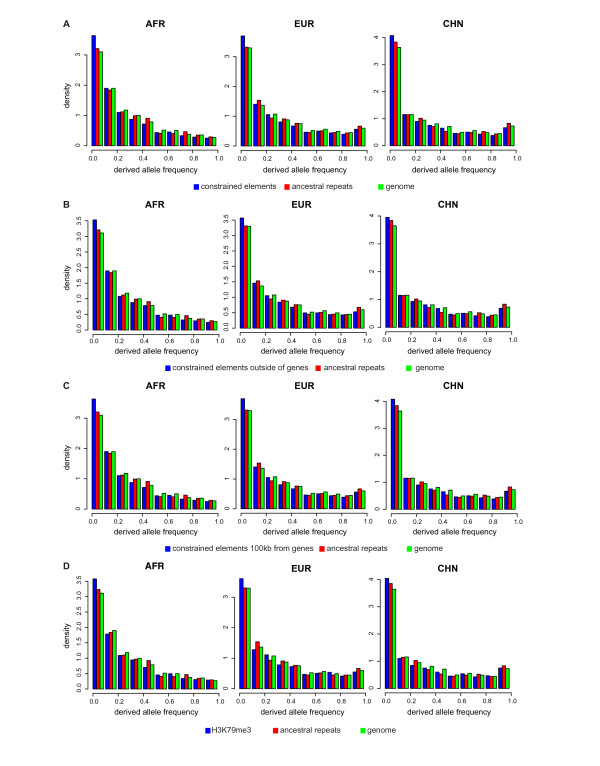
**Histograms illustrating the distribution of the derived allele frequencies for different SNP classes**. Data for all three Perlegen populations (AFR, EUR, and CHN) are presented for the genome as a whole (green), ancestral repeats (red), and various SNP classes of functional significance (blue). Only resequenced Perlegen SNP markers are included in this analysis to minimize ascertainment bias. A) Evolutionarily constrained elements B) Constrained elements excluding genic regions C) Constrained elements excluding regions closer than 100 kb to an annotated gene D) H3K79me3 regions.

For the constrained elements, we employ an alternative approach to ensure that our observation is not solely due to constrained element SNPs localized in coding regions. We considered subsets of the SNPs in the constrained element class that are located outside of genes, at least 1 kb from the closest gene, and at least 100 kb from the closest gene. In all three Perlegen populations, the derived allele frequencies of SNPs in constrained elements outside of genes were significantly lower than the derived allele frequencies in the genome as a whole and in ancestral repeats (Figure 2B; Table 5). We observed similar results when we only considered constrained elements at least 1 kb and constrained elements at least 100 kb from the closest gene relative to the genome as a whole and ancestral repeats (Figure 2C, Table 5). In summary, classes created by placing greater restrictions on the constrained elements class still display statistical significance.

**Table 5:** Comparison of the distribution of derived allele frequencies (DAF) for SNPs within several classes.

class	Perlegen population	class DAF median	ancestral repeats		genome	
			
			DAF median	*p*-value	DAF median	*p*-value
constrained elements	AFR	0.174	0.196	3.6 × 10^-5^	0.205	< 1 × 10^-8^
	
	EUR	0.188	0.208	3.4 × 10^-4^	0.229	< 1 × 10^-8^
	
	CHN	0.174	0.208	8.7 × 10^-3^	0.217	< 1 × 10^-8^

constrained elements 1 kb from genes	AFR	0.174	0.196	3.2 × 10^-3^	0.200	< 1 × 10^-8^
	
	EUR	0.190	0.208	5.4 × 10^-3^	0.229	< 1 × 10^-8^
	
	CHN	0.188	0.208	6.0 × 10^-2^	0.217	< 1 × 10^-8^

constrained elements 100 kb from genes	AFR	0.174	0.196	1.1 × 10^-2^	0.200	1.0 × 10^-8^
	
	EUR	0.205	0.208	9.6 × 10^-3^	0.229	1.1 × 10^-6^
	
	CHN	0.188	0.208	8.3 × 10^-2^	0.217	8.5 × 10^-7^

constrained elements outside of genes	AFR	0.174	0.196	3.0 × 10^-3^	0.196	< 1 × 10^-8^
	
	EUR	0.188	0.208	5.3 × 10^-3^	0.229	< 1 × 10^-8^
	
	CHN	0.188	0.208	6.1 × 10^-2^	0.217	< 1 × 10^-8^

H3K79me3	AFR	0.174	0.196	8.0 × 10^-3^	0.200	< 1 × 10^-8^
	
	EUR	0.208	0.208	1.6 × 10^-2^	0.229	1.7 × 10^-7^
	
	CHN	0.188	0.202	1.2 × 10^-1^	0.217	5.0 × 10^-8^

Classes presented are 1) constrained elements, 2) constrained elements at least 1 kb from the closest gene, and 3) constrained elements at least 100 kb from the closest gene, 4) constrained elements outside of genes, and 5) H3K79me3 regulatory attributes in comparison with the genome as a whole and ancestral repeats. We perform a Mann-Whitney U-test to compare the DAF distribution for SNPs in constrained elements, constrained elements outside of genes, and H3K79me3 regulatory attributes with that of the genome and that of ancestral repeats. We list the resulting p-values as well as the median DAF for each class.

As mentioned above, several classes derived from biological experiments have strong evidence of negative selection. For example, the derived allele frequencies in SNPs in regions known to have specific histone modifications had lower derived allele frequencies than SNPs in the genome as a whole or in ancestral repeats. Chief among these was H3K79me3 (Figure 2D; Table 5). This difference was statistically significant in all three populations when compared to the genome and in two of the populations when compared to ancestral repeats (Table 5).

## Discussion

Here we have shown that SNPs in several classes of genomic elements appear to be under negative selection. Most notably, we find that SNPs in constrained elements, even when 100 kb from the nearest gene, have a lower derived allele frequency than SNPs in presumably neutrally evolving regions. The constrained elements represent regions of the genome that are highly conserved among multiple species. Since it is thought that most SNPs arose subsequent to the last common ancestor of chimpanzees and humans, cross-species conservation in a region does not imply that the region is intolerant of new SNPs in humans. However, our work shows that an analysis of SNPs in human populations definitively demonstrates significant negative selection in these regions. Furthermore, since we find evidence for negative selection when the constrained elements are restricted to large distances from the closest annotated gene, we purport that constrained regions provide a meaningful class of putatively functionally important SNPs unto themselves without any contributions from known genes. These observations are in agreement with the findings of other groups that focused specifically on conserved regions rather than the broader approach we pursue [35-37].

Regulatory attributes represent another potentially useful group of classes for identifying functional SNPs. Our analysis finds two regulatory attributes, H3K79me3 and H3K36me3, with substantial support for negative selection. These attributes annotate regions of the genome where methylation of two specific lysines in histone H3 have been detected. The covalent addition of methyl groups to histones has been associated with both gene silencing and expression. The durability of the bond allows for epigenetic changes in gene expression that can be passed on to daughter cells. Whole genome scans of methylation sites in human cells revealed that many H3K36me3 methylations occur within transcribed regions of highly active genes [44,45]. Because this methylation peaks near the 3' end of active genes it has been hypothesized to play a role in RNA termination [46]. Whereas the H3K79me3 methylation is less well defined, several groups report a modest correlation with both highly expressed and silenced genes [44,47]. Regardless of their specific function, we believe that these regulatory attributes represent a refinement over a predicted promoter class since they are experimentally derived and displayed significant evidence of selection despite their small sample size.

In addition, other regulatory classes such as miRanda elements have some evidence of negative selection as well (Tables 2 and 3). miRanda is open-source software that predicts targets for microRNA-mediated translational repression in the 3' untranslated region of genes [48,49]. To date, the miRanda algorithm has identified 1,934,522 putative binding targets in 31,869 human gene isoforms for 677 currently known human microRNAs [50]. Although the set of rules for target prediction are not identical, our findings regarding microRNA binding targets concur with those of Chen et al. [29]. The miRanda algorithm incorporates current biological knowledge on target rules and relies on interspecies conservation. The miRanda class is distinctively different from the constrained elements as it does not apply the GERP scoring algorithm to the multiple sequence alignment across the same ten species. In addition, these regions represent an entity whose function is biologically distinct. However, it is not surprising that a class such as the miRanda class, which relies on interspecies conservation, shows evidence of negative selection. Overall, our analysis highlights specific regulatory elements that appear to have a greater potential to harbor functional SNPs.

Compared to the vast majority of classes we investigate, the individual regulatory elements contain relatively small sample sizes (Additional File 1). As with any statistical test, these small sample sizes negatively affect the power to detect a significant result. Even so, the miRanda elements and mRNA polymerase binding site (PolII), containing less than 300 and 1000 SNPs, respectively, show significant results when compared to the genome and borderline significant results when compared to ancestral repeats. Given that power is a function of sample size and magnitude of effect, in these cases a significant result suggests a substantial effect. Many of the other regulatory and non-protein coding RNA related classes are under powered due to their very small class membership (sample size < 50) and may be shown to exhibit significant negative selection in future analysis with an enlarged sample. In contrast, other classes with substantial sample sizes such as CTCF, which contains almost 12,500 SNPs, are not significant in any analysis. Thus, the identification of significant classes does not simply follow linearly with increasing sample size.

Applying the information garnered from our analysis to address the problem of an unwieldy number of SNPs to evaluate in genome-wide association studies represents an additional challenge. Our analysis provides the foundation for a comprehensive SNP prioritization scheme to select markers meriting follow-up. Such follow-up includes replicating association results in independent cohorts as well as searching for causal variants in linkage disequilibrium with associated signals. Our data can also be used to weight specific association tests in the initial stage of a genome-wide association study. Several researchers have proposed approaches to weighting SNPs in genome-wide studies using biological information such as we present here. One strategy utilizes a Bayesian approach to weighting the hypotheses in an FDR framework [51-53]. Another approach relies on hierarchical modeling, which allows for multiple sources of prior information without prejudging their value [54,55]. A third approach involves establishing groups of SNPs based on prior information and weighting them to optimize the average power of the study [56]. However, additional research is needed to determine the most powerful method. Regardless of the method, we strongly advocate harnessing the scientific insights we have found regarding genomic elements under negative selection to appropriately target regions most likely to harbor functional variants.

These data also have implications beyond the realm of genome-wide association studies. With the advent of next generation sequencing technologies, the affordability of large-scale genomic sequencing projects is rapidly increasing [57,58]. Ventures like the 1000 Genomes Project promise to provide whole-genome sequence for a large and diverse set of individuals [59]. Whole-genome sequencing has been used to identify both tumor-specific somatic mutations [60] and germline mutations in a Charcot-Marie-Tooth syndrome patient [61]. While these initial whole-genome resequencing studies focused on coding regions of annotated genes, a prioritization scheme could be employed as an efficient alternative to search for functional single nucleotide variants (SNVs) outside of coding regions. Since whole-genome sequencing will produce even more overwhelming amounts of data, the need to prioritize variants for follow-up will elevate in importance.

We expect in the future that many functional variants will be elucidated outside of the coding regions of known genes. In order to efficiently design studies to follow-up genome-wide association and whole genome sequencing efforts, we advocate a comprehensive prioritization scheme for variants based on evidence of negative selection. Our findings here illustrate the importance of considering genetic elements that lie outside of known protein-coding regions and highlight genomic elements which are most likely to contain variants that play a role in disease.

## Conclusions

These data demonstrate that SNPs outside of coding regions, especially in evolutionarily conserved regions and in putative regulatory elements, appear to be under negative selection. Some such SNPs may have physiological consequences and be responsible for human phenotypic variation. These putative functional SNPs may be a good set of SNPs to examine first when trying to find the underlying mutation responsible for observed genetic associations.

## Methods

We define classes based on the annotations available in the Ensembl database. Specifically, we access the annotations available in the Ensembl database through the Ensembl Perl application programming interface (API). This method allows us to extract all SNPs in specific regions relative to the various genome features, such as exons, transcripts, and genes, available through the database. We generate classes based on gene structure, conservation across species, and regulatory elements. For all of our analyses, we exclude SNPs which map to multiple positions in the human genome, map to the Y chromosome, or that do not appear in dbSNP. We retain SNPs mapping to alternative chromosome constructions or supercontigs if in addition they map to a single site on human chromosomes 1-22 or X.

### Gene Structure Based Classes

We utilize the annotations from the Ensembl database (release 45) to locate the regions associated with each gene structure class relative to Ensembl known protein-coding genes. These classes include promoter, splice control, and coding regions. We define promoter regions as ranging from 1000 basepairs upstream of the transcription start site to 200 basepairs downstream of the transcription start site. We define splice control regions at intron/exon interfaces (intron upstream of interface/exon downstream of interface) as ranging from 50 basepairs upstream of the interface to 2 basepairs downstream of the interface and at exon/intron interfaces (exon upstream of interface/intron downstream of the interface) as ranging from 3 basepairs upstream of the interface to 6 basepairs downstream of the interface. This splice control region definition includes the polypyrimidine track and is based on the splice control consensus sequence in humans and related mammals. In addition, we utilize the Ensembl annotations available to locate non-coding RNA genes. Specifically, we generate classes for microRNA (miRNA), small nuclear RNA (snRNA), small nucleolar RNA (snoRNA), ribosomal RNA (rRNA), transfer RNA (tRNA), small cytoplasmic RNA (scRNA), and other miscellanous types of RNA (miscRNA).

### Conservation Based Class

We utilize the results in Ensembl from the GERP scoring algorithm [62] to determine constrained elements which represent regions in the genome with a very high level of sequence conservation among the ten species (*Mus musculus, Canis familiaris, Monodelphis domestica, Rattus norvegicus, Homo sapiens, Pan troglodytes, Gallus gallus, Bos taurus, Ornithorhynchus anatinus, Macaca mulatta*) included in the multiple sequence alignment.

### Regulatory Elements Classes

We utilize the annotations from the Ensembl database (release 47) to locate regulatory features, based on experimental evidence from genome-wide assays, and the underlying regulatory attributes used to construct them [44,63]. Ensembl contains annotations for 24 distinct regulatory attributes. In addition, we utilize the Ensembl annotations available to locate cisRED [64] and miRanda [50] regulatory elements.

### Distribution of Derived Allele Frequencies within Each Class

To examine these classes for evidence of negative selection, we investigate the distribution of the derived allele frequencies (DAF) within each class. For each SNP, we find the orthologous base position in chimpanzee [29]. We define the "ancestral allele" as the chimpanzee allele equivalent in humans and the "derived allele" as the other human allele. In the event that the chimpanzee allele does not match either human allele, we compare the human alleles to the rhesus macaque reference sequence instead. For those SNPs where neither the chimpanzee nor the rhesus macaque allele matches one of the human alleles, we discard the SNP from the study. As a source for the allele frequencies, we use the publicly available genotype data from the Perlegen project populations--African Americans (AFR), European Americans (EUR), and Han Chinese from the Los Angeles area (CHN) [43]. We then compare the DAF distributions from the classes directly by employing a Mann-Whitney U-test as implemented by the software package R [65]. Specifically, we compare the DAF distributions for each class with that of the genome as a whole to detect purifying selection. As a confirmation, we repeat the comparison using the DAF for ancestral repeats, a widely accepted model of neutral selection, in place of the genome [38-42]. We define ancestral repeats using the method of Paten et al., which compares repeated sequence in five mammals (human, mouse, rat, dog, and cow), without consideration of ancestral repeat correspondence [66]. If the derived allele frequency spectrum in the class in question is lower than the derived allele frequency spectrum of the genome (or ancestral repeats), the DNA sequence in this class may be under negative selective pressure. While this approach is robust to mutation rate heterogeneity, it may be sensitive to ascertainment bias between functional regions of the genome. Ascertainment bias arises because in SNP discovery efforts often there is a bias towards SNPs in genes and common SNPs as these markers are located in well-studied regions of the genome or are more easily found. Although limiting our analysis to SNPs located in the HapMap ENCODE regions alleviates the ascertainment bias, sample sizes are compromised. As an alternative approach to minimize the ascertainment bias and maintain sample sizes, we limit the scope of the analysis to genome-wide SNPs that were resequenced by Perlegen [43]. Furthermore, for the DAF analyses, we consider only biallelic single base substitution SNPs and eliminate SNPs that overlap the two compared classes. Specifically, for comparisons to the genome, we eliminated the overlapping SNPs from the genome set only while for comparisons to ancestral repeats, we eliminated the overlapping SNPs from both classes in the comparison. After all comparisons, we determine statistical significance adjusting for the multiplicity of testing by applying the False Discovery Rate (FDR) correction with α = 0.05 [67]. We correct for 32 tests for comparisons to the genome as a whole while we correct for 44 tests for comparisons to ancestral repeats. The discrepancy in the number of tests occurs because of the necessity to use an alternative algorithm to compute accurate Mann-Whitney U-test *p*-values when one of the two classes contains a small number of samples. While this approach works efficiently when the second class contains an intermediate number of samples (ancestral repeats class), it is computationally infeasible when the second class contains a very large number of samples (genome as a whole). Thus, we eliminate these comparisons from our analysis.

### Hitchhiking Effect

We next explored the possibility that our observations are the result of an underlying hitchhiking effect or background selection. For each H3K79me3 regulatory attribute, we use the information concerning the size of the attribute and its distance to the closest annotated gene to define a region that is of identical size and distance from a randomly selected gene and, subsequently, mine this region for all SNPs. We pool the SNPs collected in this manner for all H3K79me3 regulatory attributes annotated in Ensembl and then perform two Mann Whitney tests on their DAFs against 1) the DAFs for the SNPs in the genome as a whole and 2) the DAFs for the SNPs in ancestral repeats. For these tests, we use the AFR Perlegen population to determine the frequency for each derived allele. We repeat this procedure for mining SNPs for 100 iterations and, consequently, generated 100 Mann-Whitney test *p*-values (for comparisons to the genome and 100 more *p*-values for the comparison to ancestral repeats). We compare these 100 "generated" *p*-values to the Mann-Whitney test *p*-value for the real H3K79me3 regulatory attributes and observe the number of "generated" *p*-values which are less than or equal to the "real" *p*-value. In addition, we perform this same experiment for the H3K36me3 regulatory attribute. The regulatory attributes, H3K79me3 and H3K36me3, showed the most evidence of negative selection (besides coding, nonsynonymous, and constrained element classes) and, therefore, we were most interested in ensuring these results were not due to an underlying hitchhiking effect.

## Authors' contributions

MAL designed experiments, performed all analyses, and drafted the manuscript. RJK conceived of the study, designed experiments, interpreted results, and revised the manuscript. Both authors read and approved the final manuscript.

## Supplementary Material

Additional file 1 **Evidence for negative selection among various annotation classes**. Comparison of derived allele frequency for all classes against both ancestral repeats and the whole genome is shown for all three Perlegen populations. Yellow shaded classes are statistically significant when we apply an FDR correction with α = 0.05. Only resequenced Perlegen SNP markers are included in this analysis to minimize ascertainment bias. The file can be viewed with Microsoft Excel. Click here for file
