# Supplementary material for: Predicting functionally important SNP classes based on negative selection
Source: BMC Bioinformatics. 2011 Jan 19;12:26. doi: 10.1186/1471-2105-12-26 (PMC3033802; doi:10.1186/1471-2105-12-26)
Supplement: Additional file 1 — Evidence for negative selection among various annotation classes. Comparison of derived allele frequency for all classes against both ancestral repeats and the whole genome is shown for all three Perlegen populations. Yellow shaded classes are statistically significant when we apply an FDR correction with α = 0.05. Only resequenced Perlegen SNP markers are included in this analysis to minimize ascertainment bias. The file can be viewed with Microsoft Excel. [file 1471-2105-12-26-S1.PDF]

		Genome									Ancestral Repeats								
		AFR			EUR			CHN			AFR			EUR			CHN		
class name	full sample size	rank	*p*-value	q-value	rank	*p*-value	q-value	rank	*p*-value	q-value	rank	*p*-value	q-value	rank	*p*-value	q-value	rank	*p*-value	q-value
coding	11626	1	<1.00E-08	0.00156	1	<1.00E-08	0.00156	1	<1.00E-08	0.00156	4	0.00116	0.00455	6	0.00869	0.00682	7	0.0637	0.00795
nonsynonymous	5391	2	<1.00E-08	0.00313	2	<1.00E-08	0.00313	2	<1.00E-08	0.00313	1	5.00E-08	0.00114	1	2.96E-05	0.00114	1	0.00154	0.00114
constrained elements	26146	3	<1.00E-08	0.00469	3	<1.00E-08	0.00469	3	<1.00E-08	0.00469	2	0.000036	0.00227	2	0.00034	0.00227	3	0.0087	0.00341
constrained elements minus coding	21178	4	<1.00E-08	0.00625	4	<1.00E-08	0.00625	4	<1.00E-08	0.00625	3	0.000135	0.00341	3	0.00064	0.00341	4	0.0171	0.00455
constrained elements minus genes	9192	5	<1.00E-08	0.00781	5	<1.00E-08	0.00781	5	<1.00E-08	0.00781	5	0.00299	0.00568	4	0.00533	0.00455	6	0.0606	0.00682
constrained elements 1kb from genes	8945	6	<1.00E-08	0.00938	6	<1.00E-08	0.00938	6	<1.00E-08	0.00938	6	0.00323	0.00682	5	0.00544	0.00568	5	0.0599	0.00568
regulatory features extended	51099	7	<1.00E-08	0.0109	8	3.00E-08	0.0125	7	<1.00E-08	0.0109	21	0.303	0.0239	21	0.17	0.0239	24	0.495	0.0273
H3K36me3	18479	8	<1.00E-08	0.0125	7	<1.00E-08	0.0109	8	<1.00E-08	0.0125	10	0.0401	0.0114	11	0.0394	0.0125	15	0.224	0.017
H3K79me3	7185	9	<1.00E-08	0.0141	9	1.70E-07	0.0141	9	5.00E-08	0.0141	7	0.00804	0.00795	8	0.0158	0.00909	11	0.122	0.0125
constrained elements 100kb from genes	4718	10	1.00E-08	0.0156	10	1.09E-06	0.0156	10	8.50E-07	0.0156	8	0.0109	0.00909	7	0.00957	0.00795	9	0.0826	0.0102
splice site	4597	11	0.000796	0.0172	15	0.0246	0.0234	11	0.000768	0.0172	15	0.117	0.017	18	0.158	0.0205	17	0.26	0.0193
DnaseI	18576	12	0.00454	0.0188	11	0.00197	0.0172	12	0.00101	0.0188	22	0.347	0.025	22	0.185	0.025	25	0.525	0.0284
H3K4me3	9283	13	0.00513	0.0203	18	0.0652	0.0281	14	0.00998	0.0219	20	0.289	0.0227	23	0.255	0.0261	26	0.533	0.0295
H3K4me2	1241	14	0.00861	0.0219	14	0.0243	0.0219	15	0.0113	0.0234	12	0.0709	0.0136	14	0.068	0.0159	14	0.161	0.0159
PolII	943	15	0.011	0.0234	16	0.03	0.025	13	0.00285	0.0203	11	0.0632	0.0125	13	0.0631	0.0148	8	0.0745	0.00909
miRanda	289	16	0.0149	0.025	20	0.115	0.0313	17	0.0299	0.0266	9	0.0343	0.0102	17	0.133	0.0193	10	0.0903	0.0114
cisRED	1014	17	0.0245	0.0266	12	0.0191	0.0188	16	0.0158	0.025	13	0.105	0.0148	12	0.051	0.0136	13	0.158	0.0148
H2BK5me1	338	18	0.061	0.0281	19	0.107	0.0297	19	0.0434	0.0297	14	0.11	0.0159	16	0.121	0.0182	12	0.148	0.0136
H3K4me1	1674	19	0.0922	0.0297	13	0.0229	0.0203	20	0.0507	0.0313	19	0.262	0.0216	15	0.0765	0.017	19	0.353	0.0216
regulatory features core	25450	20	0.126	0.0313	17	0.051	0.0266	18	0.0355	0.0281	27	0.55	0.0307	24	0.331	0.0273	28	0.697	0.0318
H3K27me1	181	21	0.128	0.0328	26	0.712	0.0406	27	0.65	0.0422	17	0.161	0.0193	30	0.634	0.0341	30	0.712	0.0341
H3K9me1	775	22	0.143	0.0344	24	0.598	0.0375	21	0.0717	0.0328	18	0.25	0.0205	29	0.571	0.033	18	0.273	0.0205
promoter	7154	23	0.421	0.0359	25	0.641	0.0391	25	0.568	0.0391	28	0.567	0.0318	28	0.513	0.0318	33	0.819	0.0375
H3R2me1	63	24	0.467	0.0375	21	0.17	0.0328	22	0.163	0.0344	24	0.493	0.0273	19	0.168	0.0216	16	0.245	0.0182
H4K20me1	785	25	0.645	0.0391	27	0.774	0.0422	29	0.832	0.0453	30	0.685	0.0341	32	0.709	0.0364	36	0.911	0.0409
H2AZ	1596	26	0.702	0.0406	23	0.387	0.0359	24	0.367	0.0375	31	0.726	0.0352	26	0.403	0.0295	27	0.668	0.0307
all RNA genes	97	27	0.747	0.0422	22	0.175	0.0344	23	0.315	0.0359	32	0.774	0.0364	20	0.169	0.0227	23	0.406	0.0261
H3K36me1	87	28	0.78	0.0438	28	0.847	0.0438	31	0.946	0.0484	34	0.795	0.0386	36	0.828	0.0409	40	0.96	0.0455
H3K27me3	956	29	0.788	0.0453	29	0.889	0.0453	28	0.655	0.0438	33	0.784	0.0375	34	0.81	0.0386	34	0.821	0.0386
H4K20me3	883	30	0.943	0.0469	30	0.893	0.0469	26	0.578	0.0406	40	0.92	0.0455	37	0.831	0.042	31	0.768	0.0352
H3K9me3	2243	31	0.95	0.0484	31	0.987	0.0484	30	0.924	0.0469	37	0.897	0.042	38	0.902	0.0432	38	0.944	0.0432
CTCF	12486	32	0.996	0.05	32	0.997	0.05	32	0.991	0.05	38	0.9	0.0432	35	0.823	0.0398	39	0.957	0.0443
H3R2me2	20	33	not computed		33	not computed		33	not computed		41	0.947	0.0466	41	0.964	0.0466	42	0.975	0.0477
H3K9me2	14	34	not computed		34	not computed		34	not computed		35	0.828	0.0398	42	0.969	0.0477	32	0.77	0.0364
H4R3me2	28	35	not computed		35	not computed		35	not computed		43	0.975	0.0489	43	0.984	0.0489	41	0.972	0.0466
H3K27me2	13	36	not computed		36	not computed		36	not computed		39	0.914	0.0443	39	0.942	0.0443	35	0.89	0.0398
H3K79me1	28	37	not computed		37	not computed		37	not computed		42	0.95	0.0477	44	0.987	0.05	44	0.996	0.05
H3K79me2	5	38	not computed		38	not computed		38	not computed		25	0.505	0.0284	25	0.396	0.0284	20	0.362	0.0227
miRNA	10	39	not computed		39	not computed		39	not computed		29	0.623	0.033	27	0.405	0.0307	22	0.383	0.025
miscRNA	32	40	not computed		40	not computed		40	not computed		23	0.41	0.0261	10	0.0279	0.0114	21	0.362	0.0239
rRNA	26	41	not computed		41	not computed		41	not computed		44	0.988	0.05	40	0.963	0.0455	43	0.983	0.0489
snRNA	8	42	not computed		42	not computed		42	not computed		26	0.512	0.0295	31	0.654	0.0352	29	0.711	0.033
snoRNA	22	43	not computed		43	not computed		43	not computed		16	0.153	0.0182	9	0.0245	0.0102	2	0.00384	0.00227
tRNA	11	44	not computed		44	not computed		44	not computed		36	0.831	0.0409	33	0.755	0.0375	37	0.917	0.042
